# Accelerating microfluidic capillary electrophoresis-mass spectrometry for charge-variant and glycoform analysis of intact monoclonal antibodies

**DOI:** 10.1007/s00216-026-06374-9

**Published:** 2026-02-05

**Authors:** Ruben Cageling, Erin A. Redman, J. Scott Mellors, Karin Lubbers-Geuijen, Govert W. Somsen, Kevin Jooß

**Affiliations:** 1 Protein Purification & Characterization Department, Polpharma Biologics Utrecht, Yalelaan 46, 3584 CM Utrecht, The Netherlands; 2 Division of BioAnalytical Chemistry, Department of Chemistry and Pharmaceutical Sciences, Vrije Universiteit Amsterdam, de Boelelaan 1085, 1081 HV Amsterdam, The Netherlands; 3 Centre for Analytical Sciences Amsterdam (CASA), Amsterdam, The Netherlands; 4 Repligen Corporation, Morrisville, NC USA; 5 Move Analytical LLC, Carrboro, NC USA

**Keywords:** Capillary zone electrophoresis, Microfluidic chip, Charge heterogeneity, Glycoforms, High throughput

## Abstract

The analytical determination of critical quality attributes (CQAs) of monoclonal antibodies (mAbs) is often time-consuming. Hence, increasing analysis throughput can shorten biopharmaceutical development timelines and enables rapid decision-making. Microfluidic capillary electrophoresis coupled to mass spectrometry (MCE-MS) allows fast analysis of charge variants and glycoforms of mAb samples. In a recently published MCE-MS method, we used a microfluidic chip with a 22-cm separation channel length, permitting analysis of 96 samples per day. To further increase the throughput of the method and better meet the needs of the biopharmaceutical industry, we propose the use of a chip with a distinctly shorter channel length (10 cm). However, a substantial drop in electrophoretic resolution was observed due to the increased laminar flow caused by the pressure put on the shortened channel during analysis. To mitigate this phenomenon, the MCE setup was modified to allow independent adjustment of the gas pressure on the separation channel inlet. The mAb charge-variant resolution significantly improved by reducing the inlet pressure from 2.0 to 0.75 psi. The performance of the modified setup was assessed by analysis of mAb samples from cell lines, mimicking a typical biosimilar clone screening. The original (22-cm channel, 2.0 psi on inlet) and modified (10-cm channel, 0.75 psi on inlet) setups yielded comparable separation profiles and number of MS-identified proteoforms across all clone mAbs. Overall, the new MCE-MS method reduces total analysis time per sample by 3.3-fold, which conceptually facilitates the analysis of more than 300 samples per day with minimal loss in separation performance.

**Graphical abstract:**

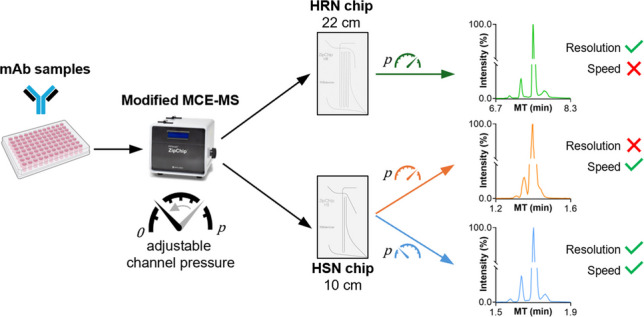

**Supplementary Information:**

The online version contains supplementary material available at 10.1007/s00216-026-06374-9.

## Introduction

Biopharmaceuticals have developed into an indispensable class of medicines for the treatment of serious diseases such as cancer, autoimmune diseases, and blood disorders [1]. The majority of biopharmaceuticals are monoclonal antibodies (mAbs), but other antibody-based formats such as bispecific antibodies (BsAbs), Fc-fusion proteins, and antibody fragments are also emerging [1, 2]. The popularity of these antibody-based drugs can be explained by their well-understood behavior and engineering, which facilitates the development of new therapies [1]. Each year, an increasing number of novel biotherapeutics are entering the market and, in the meantime, some early available biopharmaceuticals lose patent protection. Once the patent of the originator drug expires, the market opens for generic alternatives of the originator known as biosimilars, which largely resemble the originator product with only minor deviations in efficacy, safety, and quality allowed [2, 3]. As a growing number of novel and generic antibody-based medicines are commercialized, the biopharmaceutical market is becoming increasingly competitive, with the cost-effectiveness of production and development of biotherapeutics regarded as a critical factor. Therefore, biopharmaceutical companies are seeking routes to optimize and accelerate their processes.

One important factor of biotherapeutic development that can be expedited is the analytical characterization of biological and physico-chemical properties of the protein of interest. These properties are referred to as critical quality attributes (CQAs), as they can affect the safety and efficacy of the drug [2, 3]. Two of the most frequently evaluated CQAs of mAbs are charge variants and the glycosylation profile. For instance, the glycan composition can influence the mechanism of action and the pharmacokinetics and/or pharmacodynamics of a mAb [4]. Besides glycosylation, other post-translational modifications (PTMs) of the protein are vital to monitor [5, 6]. Some PTMs, such as asparagine deamidation and C-terminal lysine clipping, can alter the net charge of a protein. These charge-differentiating proteoforms can be separated using charge-variant assays (CVAs), such as ion-exchange chromatography (IEX) and capillary zone electrophoresis (CZE) [5, 6]. When these separation techniques are coupled with MS, the identity of the mAb charge variants and the glycoform composition can be determined from the same analysis [7–9]. Therefore, IEX-MS or CZE-MS are attractive approaches to measure glycosylation and the charge-variant profiles of a mAb simultaneously. However, CVA methods used in industry are often not MS compatible, commonly encompass long analysis times (> 15 min per sample), and require high sample volumes, which is a disadvantage in early development stages that typically involve large sample numbers, low protein titers and volumes, and short decision timelines. For instance, this is the case in the cell line generation stage of biosimilar development, where > 200 samples can be expected after single cell seeding in the deep well plates. In this phase, a decision on which cell lines to proceed is required within days while cells are still in culture [3, 10, 11]. If CQAs could be analyzed earlier in the development phase, aberrant cell lines can be identified earlier in the development (e.g., in the stable pool phase), which ultimately aids in deciding if a biosimilar program is feasible. Hence, early detection of non-feasible programs can significantly reduce costs in biopharmaceutical development. However, as noted by Clarke et al*.*, product quality analysis is typically not performed in early cell line development, as biopharmaceutical companies believe that traditional analytical methods would increase total development time [10]. Consequently, earlier CQA analysis in biopharmaceutical clone selection would require highly sensitive and rapid analytical techniques. In this context, there is an ongoing effort in the analytical community to speed up separation methods for biopharmaceutical characterization [11, 12].

CZE-MS is considered an attractive methodology for performing CVA in biotherapeutic development due to its MS compatibility, high sensitivity, and low volume requirement. Microfluidic CE-MS (MCE-MS), where CZE is performed on a microfluidic glass chip in etched channels, enables efficient and robust nano electrospray ionization (nanoESI) facilitated by an integrated emitter in the corner of the chip [13, 14]. MCE-MS setups offer the advantage of combining short analysis times with good separation performance. For these reasons, MCE-MS is increasingly being used in biopharmaceutical analysis as a rapid tool to quantify charge variants and identify PTMs such as glycosylation or C-terminal heterogeneity of antibody samples [7, 8, 13–19].

In our previous work, we demonstrated how MCE-MS can be employed for rapid biosimilar clone screening [8]. We utilized MCE-MS employing a so-called HRN (High-Resolution Native) chip for the separation of mAb charge variants. This chip features a 22-cm channel length, resulting in 10 to 15 min run time per sample. In the present study, we aimed to reduce analysis times even further by using an HSN (High-Speed Native) chip featuring a channel length of 10 cm, to better suit the demands of the biopharmaceutical industry. Initial tests with this chip indicated that, although migration times of the charge variants were significantly shortened, electrophoretic resolution was considerably decreased due to extra band broadening. We concluded that this broadening is likely caused by a fixed gas pressure applied to the separation channel during analysis, which induces considerable laminar flow. Therefore, the MCE-MS system was modified to enable independent control of the separation channel pressure, decoupling it from the other pressure lines in the system. Subsequently, we studied the impact of the magnitude of this pressure on the resolution of charge variants of NISTmAb employing the HSN chip and made comparisons with results obtained with the HRN chip using the original MCE-MS settings. To evaluate the overall performance of the optimized method using the HSN chip, we further analyzed representative mAb samples from production cell lines by mimicking a typical mAb biosimilar cell line screening approach on a smaller scale. These results were benchmarked against our previous MCE-MS method employing the HRN chip. The goal of the study was to improve the throughput of mAb analysis with MCE-MS without compromising the selectivity and sensitivity.

## Materials and methods

### Materials

ZipChip Charge Variants Analysis kit (containing background electrolyte and diluent), High-Speed Native (HSN) chip, and High-Resolution Native (HRN) chip were obtained from Repligen Corporation (Waltham, MA, USA). Zeba 7 K MWCO spin cartridges were purchased from Fisher Scientific (Landsmeer, The Netherlands). NISTmAb was purchased from Sigma-Aldrich (Amsterdam, The Netherlands). Four early-stage biosimilar candidate mAbs, designed after the same originator mAb, were generated in cell lines in-house. The corresponding originator mAb was sourced from the market.

### Sample preparation

For the pressure evaluation study, NISTmAb was diluted in ZipChip Charge Variants Analysis Diluent to a final concentration of 250 µg/mL. For the biosimilar clone screening study, four cell lines were transfected with DNA corresponding to the amino acid sequence of an IgG1 biosimilar candidate. The four transfected cell lines were then subjected to a fed-batch cell culture. After the harvest of the cell cultures, a sample was taken from each cell line, which resulted in four samples in total (referred to as clones 1 to 4). Both the originator mAb and the harvested samples were protein A-purified using a method outlined in a previous study [8]. After protein A purification, the samples were buffer exchanged into ZipChip CVA diluent using Zeba 7 K MWCO spin cartridges and subsequently diluted to 250 µg/mL with ZipChip CVA diluent. All samples were measured in triplicate.

### MCE-MS analysis

**Fig. 1:**
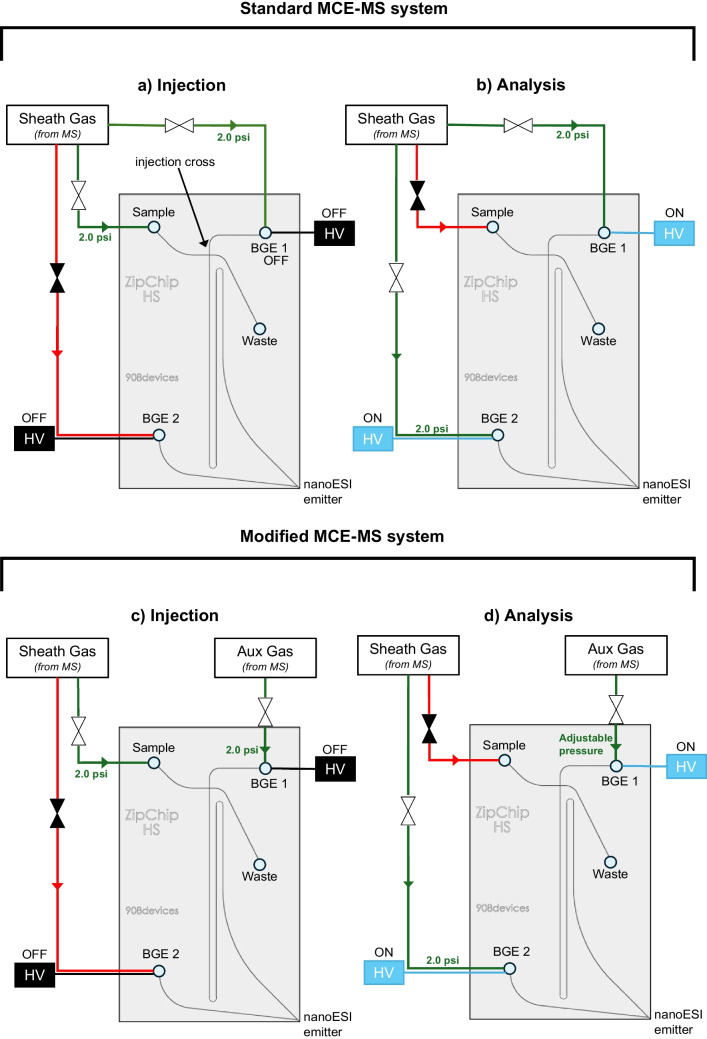
Schematics showing the flow channels, gas and high-voltage lines, and annotated wells of a “High-Speed Native” (HSN) microfluidic chip for MCE-MS. Red and green lines indicate gas lines connected to outlets on the mass spectrometer. Black and blue lines indicate high-voltage connections (denoted HV). **a**, **b** The injection and analysis settings, respectively, for the standard MCE-MS system. For sample injection (**a**), a pressure of 2.0 psi is applied on both the “Sample” and “BGE 1” wells for a certain time, introducing a sample plug via the injection cross into the separation channel, while part of the sample goes to waste. For analysis (**b**), the pressure valve on the gas line to the “Sample” well is closed and HV is applied across the separation channel. At the same time, the valve in the gas line to the “BGE 1” well remains open and the valve in the gas line to the “BGE 2” well is opened. These settings provide a constant pressure of 2.0 psi to the separation channel during analysis and a constant flow of sheath liquid towards the nanoESI emitter. **c**, **d** The injection and analysis settings, respectively, for the modified MCE-MS system. The “BGE 1” well has a separate gas pressure line connected to the Aux gas outlet of the mass spectrometer. For sample injection (**c**), a pressure of 2.0 psi is applied on both the “Sample” and “BGE 1” wells for a certain time. For analysis, the pressure valve on the gas line to the “Sample” well is closed and HV is applied across the separation channel. At the same time, the valve in the gas line to the “BGE 2” well is opened, providing sheath liquid at 2.0 psi towards the nanoESI emitter, while the valve in the gas line to the “BGE 1” well remains open and an adjustable pressure is applied

A ZipChip MCE-MS (Repligen Corporation, Waltham, MA, USA) system with adapted gas lines was utilized in conjunction with an HSN (High-Speed Native) chip or a HRN (High-Resolution Native) chip. The HSN chip (Fig. 1) features a separation channel with a length of 10 cm, a width of 70 µm, and a depth of 10 µm. The channel was covalently coated with a neutral polymer. The following settings for the MCE-MS for the HSN chip were used: field strength, 1000 V/cm; injection volume, 1.5 nL; pressure assist enabled at 0.0 min; method run time (excluding BGE refresh and injection step), 3 min. This method is further referred to as the HSN setup. The previously developed CVA MCE-MS method employing an HRN (High-Resolution Native) chip was used for benchmarking the HSN setup. The HRN chip has a 22-cm separation channel; coating and further dimensions are the same as for the HSN chip. The HRN setup employed the following settings: field strength, 500 V/cm; injection volume, 1.0 nL; pressure assist enabled at 0.0 min; method run time, 10 min. Both MCE-MS methods utilized the ZipChip Charge Variants Analysis background electrolyte (CVA BGE). This BGE is also used as a sheath liquid.

The MCE instrument was coupled to an Exploris 240 Orbitrap mass spectrometer (Thermo Scientific, Bremen, Germany) by replacing the ESI source housing with the MCE interface. For all measurements, the mass spectrometer was operated in Intact Protein mode with standard trapping pressure. The ion transfer tube was set at 300 °C and full scan data were acquired with the following settings: resolution, 30,000; mass range, 2000–8000 *m*/*z*; RF lens, 150%; normalized AGC target, 100%; max injection time, 10 ms; microscans, 2; source fragmentation, 135 V. The standard MCE instrument uses the sheath gas nitrogen line, which is normally used by the mass spectrometer to assist in spray nebulization during ESI and reroutes it to the “BGE 1,” “BGE 2,” and “Sample” wells of the microfluidic chip (Fig. 1a, b). For the HRN setup, the sheath gas pressure was set to 2.0 psi. The HSN setup was performed on a modified MCE-MS system, where the Aux gas nitrogen line of the mass spectrometer was connected to the “BGE 1” well (Fig. 1c, d). The HSN setup utilized the time-dependent gas control program of the mass spectrometer. The pressures at the start of the analysis were set to 2.0 psi and 4.0 psi for sheath gas and Aux gas, respectively. At 0.1 min after the start of the analysis, the Aux gas pressure was lowered to a setting of 1.5 psi while the sheath gas pressure was maintained at 2.0 psi. Note that the actual pressure applied to the “BGE 1” well on the chip was approximately half of the Aux gas pressure setting (experimentally determined). So, an Aux gas pressure setting of 4.0 psi corresponds to an effective pressure of 2.0 psi. The sheath gas pressure applied to the “Sample” and “BGE 2” wells was monitored by the pressure gauge built into the ZipChip interface and was determined to be equal to the set pressure. All gas pressures reported in this study are the effective gas pressures.

### Data analysis

All data was acquired and processed with Chromeleon 7.3.2 (Thermo Scientific). For the gas pressure optimization study, the base peak electropherograms (BPEs) of NISTmAb were used to determine the migration time (*t*_m_, measured at the peak apex) and the full width at half maximum (FWHM) of the peaks observed for the charge variants. These values were used to assess the separation performance by calculating the plate number (*N*) for the basic variant 1 (*N*_*B1*_) and main peak (*N*_*M*_), and the resolution (*R*_*S*_) between basic variants 1 and 2 (*R*_*S,B2-B1*_), and basic variant 1 and the main peak (*R*_*S,B1-M*_) using Eqs. 1 and 2.

1 $$N=5.54 {\left(\frac{{t}_{\mathrm{m}}}{\mathrm{FWHM}}\right)}^{2}$$

2 $${R}_{s}=1.18 \frac{{t}_{\mathrm{m},2}- {t}_{\mathrm{m},1}}{{\mathrm{FWHM}}_{1}+{\mathrm{FWHM}}_{2}} \text{with }{t}_{\mathrm{m},2}>{t}_{\mathrm{m},1}$$

The main peak and the acidic variant were partially comigrating, which hampered accurate determination of the FWHM of the acidic variant. Therefore, the peak to valley ratio (*p/v*_*M-A*_) was used to assess the quality of this separation (Eq. 3).

3 $${p/v}_{M-A}=\frac{{H}_{\text{acidic variant}}}{{V}_{\text{acidic variant}}}$$

where

*H*_acidic variant_: is the peak height of the acidic variant peak
*V*_acidic variant_: is the height of the peak valley between the main peak and the acidic variant.

For the biosimilar clone screening study, the acquired base peak electropherograms (BPEs) were used for the calculation of the relative peak areas of the charge variants. The raw data files of the clone and the originator mAbs were exported and subjected to Sliding Window Deconvolution in Biopharma Finder 5.1 (Thermo Scientific). Deconvolution was performed using the ReSpect™ algorithm over the entire charge profile, using the following settings: output mass range, 144,000 to 150,000 Da; charge state range, 10 to 40; sequence matching mass tolerance, 30 ppm; target average spectrum width, 0.05 min (0.09 min for HRN method); scan offset, 1; merge tolerance, 30 ppm; max RT gap, 0.05 min (0.1 min for HRN method); min. number of detected intervals, 3. The assignment of proteoforms was based on the primary sequence of the originator mAb, for which the following PTMs were considered: C-terminal lysine, C-terminal proline amidation (both PTMs can be found on one or two heavy chains), carboxymethyllysine, deamidation, and oxidation. The CHO glycan library in Biopharma Finder was used for glycoform assignment. Positive proteoform and glycoform identifications were made if a specific assignment was within the intact mass tolerance of ±30 ppm. The assignments were verified based on their migration behavior relative to the main peak in the electropherogram.

For each detected proteoform, the relative abundance was calculated by dividing the MS intensity of that proteoform in the deconvoluted mass spectrum by the MS intensity of the most intense proteoform (G0F + G0F with no other modifications). Only proteoforms that were detected in all triplicates and with a relative abundance of ≥ 0.1% were reported. If a proteoform was identified in only one or two of the triplicates, the corresponding extracted ion electropherogram (EIE) of that proteoform was exported from Biopharma Finder (based on the same charge states) and used in Chromeleon to manually determine the relative abundance based on the peak area of the proteoform in the EIE for both setups. For the determination of the glycoform distribution, the fractional abundance of each glycoform was calculated by dividing the MS intensity of a glycoform by the sum of the MS intensities of all detected glycoforms. The degree of afucosylation and galactosylation were calculated as described in our previous work [8]. For the cell line selection of the four mAb biosimilar candidates, a scoring approach reported in our previous work was followed [8]. For this cell line selection, the following CQAs were considered: acidic charge variants, basic charge variants, the degree of afucosylation, and the degree of galactosylation. A weighing factor of 1 was applied for all CQAs. For each CQA, the originator range used for the scoring approach was determined based on the average outcome of the originator ± 10% (*n* = 3). All obtained results were processed in Microsoft Excel and Graphpad Prism 10.0.

## Results and discussion

### MCE-MS chip and instrument design

**Fig. 2:**
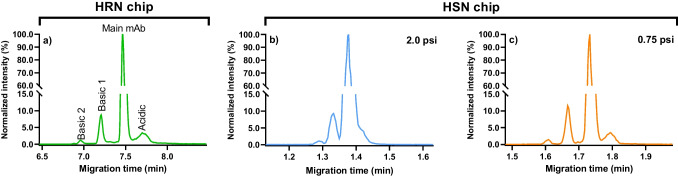
Normalized base peak electropherograms (BPEs) obtained during MCE-MS of NISTmAb using **a** the HRN chip (separation channel length of 22 cm) under default conditions (HRN setup) and **b**, **c** the HSN chip (separation channel length of 10 cm) employing a separation-channel gas pressure of **b** 2.0 psi and **c** 0.75 psi. Peaks are annotated in panel **a**

The previously developed CVA application with MCE-MS utilized an HRN chip with a 22-cm channel length, obtaining separation of charge variants in 10–15 min of run time per sample (Fig. 2a). Although such run times are relatively fast compared to other techniques such as IEX, larger sample sets (above 100 samples) as encountered in cell line screening, stability studies, or large process development studies will still take multiple days to run. To increase sample throughput, we considered the use of a chip (denoted as HSN) with a 10-cm channel length. However, when leaving the other experimental conditions unchanged, electrophoretic resolution was greatly diminished (Fig. 2b), largely due to extra peak broadening resulting in decreased plate numbers of the charge variant peaks. Peak broadening can be mitigated by increasing the BGE viscosity, but this would require drastic changes to an already optimized BGE composition, which would most likely result in a reduced separation of the charge variants. Since this would not cancel out the root cause of the band broadening, the BGE was kept the same. To understand the source of the extra band broadening, it is rational to have a closer look at the gas pressures applied by the system during injection and analysis (Fig. 1a and b) under standard conditions. For sample injection, a pressure of 2.0 psi is applied to both the “Sample” well and the “BGE 1” well for 0.1 min, while the valve in the gas line to the “BGE 2” well is closed (Fig. 1a). The applied pressure enables injection of part of the sample plug via the channel cross into the separation channel. For analysis, the pressure valve in the gas line to the “Sample” well is closed, while the valve in the gas line to the “BGE 2” well is opened and the valve in the gas line to the “BGE 1” well remains open (Fig. 1b). At the same time, high voltage is applied across the separation channel, inducing CE separation of mAb charge variants which migrate towards the nanospray emitter. The pressure on the “BGE 2” well provides a flow of sheath liquid which facilitates nano electrospray ionization and merges with the separation channel just before the emitter. In order to prevent a small counter flow of sheath liquid into the separation channel at this point, a constant pressure of 2.0 psi is maintained on the BGE 1 well during analysis. This pressure also prevents residual sample present around the channel cross from entering the separation channel during analysis. Notably, the constant pressure on the BGE 1 well induces a modest laminar flow in the separation channel towards the emitter. When the channel length is reduced, the magnitude of this pressure-induced flow will increase. Consequently, the relative contribution of the parabolic flow profile to band broadening will increase when using the HSN chip instead of the HRN chip, leading to lower plate numbers, and thus lower resolutions, as will be illustrated more elaborately in the next section. To mitigate the band broadening effect, we modified the gas lines of the MCE-MS system to allow for independent adjustment of the pressure applied to the separation channel (Fig. 1c, d). The “BGE 1” well was connected to the pressure line coming from the Aux gas source of the mass spectrometer, while the sheath gas pressure of the mass spectrometer remained connected to the “Sample” and “BGE 2” wells. This modification allows for selecting a constant pressure of choice on the separation channel while maintaining the pressure option of 2.0 psi required for the injection channel (Sample well) and auxiliary sheath liquid channel (BGE 2 well) on the chip (Fig. 1d).

### Effect of separation-channel pressure on charge-variant resolution

The impact of the gas pressure applied to the separation channel was evaluated for NISTmab as a model protein. The effective inlet pressure on the “BGE 1” well was varied between 0.5 and 2.0 psi with increments of 0.25 psi. Figure 2b and c depict the base-peak electropherograms (BPEs) of NISTmAb that were obtained with the HSN chip (10-cm channel) using effective “BGE 1” pressures of 2.0 psi and 0.75 psi, respectively. The BPEs obtained using the other tested gas pressures are shown in Figure S1. For comparison, Fig. 2a depicts the BPE of NISTmAb obtained with the HRN chip (22-cm channel) using unmodified MCE-MS settings. For all tested gas-pressure settings, the electropherograms revealed one main peak, two peaks from basic variants migrating before the main peak, and one peak from acidic variants migrating after the main peak, typical for a NISTmAb charge variant profile. The BPE obtained with the HRN setup displays sharp, well-resolved peaks, with the acidic variant almost fully separated from the main peak, which migrates at about 7.5 min (Fig. 2a). Using the HSN setup at a “BGE 1” pressure of 2.0 psi (i.e., the same pressure as used in the HRN setup), the main peak migrates at around 1.4 min (Fig. 2b), which means that the main peak migrates ~ 5.3 times faster with the HSN setup. This substantial gain in analysis speed results from the 2.2-fold shorter channel length and 2-fold increase in field strength. Interestingly, the main peak migrates even faster than the expected decrease in migration time (5.3- vs. 4.4-fold difference) due to the additional laminar flow induced by the gas pressure. However, as already indicated above, charge-variant resolution deteriorated considerably when using the HSN chip at 2.0 psi pressure. This was most notable for the peaks of the main mAb and its acidic variant, which were not properly separated under the applied conditions. Decreasing the gas pressure from 2.0 to 0.5 psi, and thus reducing the laminar flow, largely restored the resolution of the charge variants and the main peak (Figure S1 and Fig. 2c). As a consequence, migration times slightly increased at lower pressure settings. For example, the average migration time of the main peak shifts from 1.42 ± 0.05 to 1.72 ± 0.02 min when reducing the applied pressure from 2.0 to 0.5 psi (Table S1).

**Fig. 3:**
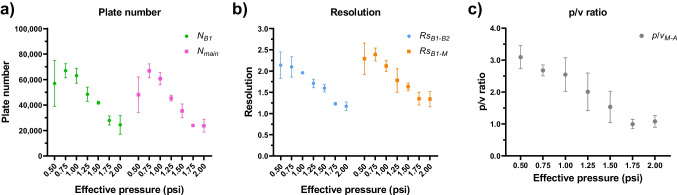
Influence of the effective gas pressure applied to the separation channel on the separation performance obtained during MCE-MS analysis of NISTmAb using the HSN chip. **a** Plate numbers of basic variant 1 (*N*_*B1*_, green circles) and the main mAb (*N*_main_, pink squares). **b** Resolutions of peak pairs basic variant 2-basic variant 1 (*R*_*S,B2-B1*_, blue circles) and basic variant 1-main mAb (*R*_*S,B1-M*_, orange squares). **c** Peak-to-valley ratio for the main mAb and acidic variant peaks (*p/v*_*M-A*_, grey circles). The error bars indicate ± 1 standard deviation (*n* = 3)

To evaluate the obtained separations more quantitatively, plate numbers for the basic variant 1 (B1) and the main peak, and resolutions for critical peak pairs of the basic variants and the main peak were calculated from the BPEs acquired with the HSN chip (Fig. 3a, b and Table S2). The plate numbers of B1 and the main peak show an ascending trend from about 23,000 up to almost 67,000 when the applied pressure was decreased from 2.0 to 0.75 psi, indicating a clear reduction of band broadening caused by the pressure flow (Fig. 3a). As a result, the peak pairs exhibited a 1.8-fold increase in resolution when the gas pressure was decreased (Fig. 3b). The resolutions obtained under the optimal pressure conditions for the HSN chip are smaller compared to those obtained with the HRN setup (3.5 and 3.8 for the B2-B1 and B1-main peak pairs, respectively), but the achieved resolutions are still well above 1.5 (baseline separation). As the acidic variant peak partially overlapped with the main peak, the FWHM for the acidic variant could not be accurately determined for the higher pressures (Table S2). Therefore, to evaluate the separation between the main peak and the acidic variant, the peak-to-valley (p/v) ratio (see section “Data analysis”) of this peak pair was calculated while employing different separation-channel pressures. The p/v ratio obtained with the HSN chip steadily increases with decreasing separation-channel gas pressure (Fig. 3c and Table S2), indicating better resolution of this peak pair at lower applied pressures. At a pressure of 0.75 psi, the p/v ratio obtained with the HSN chip (2.7) approaches the p/v ratio obtained with the HRN chip (3.4).

The extra band broadening caused by the gas pressure was minimal at 0.5 and 0.75 psi. For pressure values below 0.75 psi, the system became increasingly unstable, leading to high variation in plate numbers, resolution, and p/v values (Fig. 3 and Table S2). Furthermore, the resolution did not increase anymore when going from 0.75 to 0.5 psi. Therefore, a separation-channel gas pressure of 0.75 psi was selected for further experiments. For NISTmAb, the charge-variant profile obtained with the HSN chip under these conditions was highly similar to the profile obtained with the HRN setup employing a chip with a 22-cm channel (Fig. 2a and c). However, the total analysis time per sample (including chip conditioning and sample transfer steps) achieved with the optimal HSN setup was about 3.3-fold shorter than that attained with the HRN setup.

### Performance of modified MCE-MS system

**Fig. 4:**
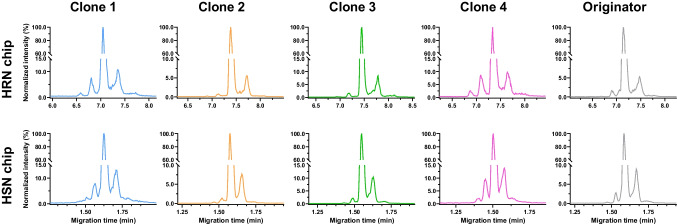
Normalized BPEs of the four clone mAbs and of the corresponding originator mAb obtained by MCE-MS using the HRN chip (upper row) and the HSN chip (bottom row) with effective separation-channel gas pressures of 2.0 and 0.75 psi during analysis, respectively

To evaluate the performance of the 10-cm channel MCE-MS system with the modified pressure arrangement, representative mAb samples were analyzed and compared with results obtained with the previously developed HRN method [8]. For this purpose, we selected a typical case of a small-scale biosimilar cell line screening in which samples were taken from four distinct cell lines expressing the same biosimilar mAb. The MCE-MS results obtained for these clone mAb samples (clones 1–4) were evaluated against the MCE-MS results obtained for the corresponding originator mAb in order to assess biosimilarity. The purified mAb samples were measured in triplicate using both the HRN chip (22-cm separation channel at 2.0 psi) and the HSN chip (10-cm separation channel at 0.75 psi). The BPEs obtained for the clones and originator mAbs are shown in Fig. 4, which shows an up to 4.4-fold decrease of peak migration times when using the optimized HSN setup. Importantly, the charge-variant separations obtained with both methods were highly similar. The resolutions achieved with the HSN chip were lower compared to the ones achieved with the HRN chip, as illustrated for the main mAb-acidic variant peak pair (Figure S2). Nevertheless, all attained resolutions were above 1.5 and thus fit for the purpose of this application.

**Fig. 5:**
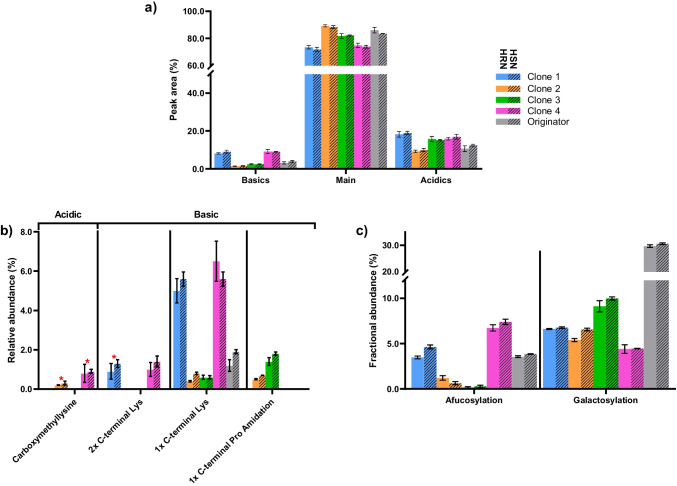
Bar charts depicting **a** the average relative peak areas (%) of the main and charge variant peaks, **b** the average relative abundance (%) of the charge-inducing proteoforms, and **c** the average fractional abundance (%) of afucosylation and galactosylation observed in the four clone mAbs and the originator mAb with MCE-MS. The solid bars show results obtained with the HRN chip with an effective separation-channel gas pressure of 2.0 psi, and the striped bars show results obtained with the HSN chip with an effective separation-channel gas pressure of 0.75 psi. **b** The following charge-inducing proteoforms and their peak of origin (in brackets) were identified: carboxymethyllysine (acidic), 2× C-terminal lysine (basic 2), 1× C-terminal lysine (basic 1), and 1× C-terminal proline amidation (basic 1). All charge-inducing proteoforms featured the glycan pair G0F + G0F and were compared to the main proteoform that contains G0F + G0F without any other PTM. An asterisk (*) denotes proteoforms that were calculated manually via EIEs. Error bars indicate ± 1 standard deviation (*n* = 3)

The peaks observed in the BPEs of the clone mAb and originator samples were integrated, and relative peak areas (%) for the basic and acidic charge variants were calculated (Fig. 5a). For each mAb sample, the relative peak areas of the basic, main, and acidic peaks obtained were highly similar for the HSN and HRN chip setup. Absolute differences in relative peak areas between the two chips were mostly within 1.0% and never higher than 2.6%. These results substantiate that the optimized HSN setup and the conventional HRN setup provide highly comparable results in terms of separation and relative analyte response. Furthermore, both setups demonstrated good relative standard deviation (RSD) for the relative peak area of each variant peak, with most RSD values well below 10%.

The acidic and basic charge variants observed in the clone and originator mAbs could be assigned to charge-inducing PTMs based on the mass spectra recorded with both MCE-MS setups (Fig. 5b). The basic charge variants migrating before the main compound were attributed to the residual presence of one or two C-terminal lysines on the mAb (mass increment, + 128 and + 256 Da, respectively), or to a single C-terminal proline amidation (–58 Da). The acidic charge variant is caused by the presence of a carboxymethyllysine (+ 58 Da), which is an advanced glycation end product (AGE) [20, 21]. In addition, the most abundant *m*/*z* found in the acidic peak of each clone mAb and the originator mAb was very close to the *m*/*z* of the main proteoform (G0F + G0F) in the main peak. This peak was tentatively attributed to asparagine deamidation (+ 0.98 Da) and/or isomerization to isoaspartic acid (no mass shift) [22, 23]. Although this PTM cannot be accurately quantified due to this minor mass shift, this CVA method is able to resolve it from the main variant because both PTMs result in acidic-migrating proteoforms. Comparable relative abundances for the specific variants were found with the HSN and HRN setups for clones 1–4 mAbs and the originator mAb.

MCE-MS not only enables assignment of charge variants but also allows extraction of detailed mass information on glycoforms. The mass spectra derived from the electropherograms of the four clone mAbs and the originator mAb were deconvoluted, and glycoforms were assigned based on their specific mass. The assigned glycoforms and their fractional abundances resulting from the HRN setup and the HSN setup are listed in Figure S3. The same glycoforms were identified by both the HSN and the HRN setup, with highly similar fractional abundances for the same glycoform attained. Based on the obtained fractional abundances of the glycoforms, the degree of galactosylation and afucosylation of the mAb was calculated, which are important CQAs for the biosimilar candidate under study (Fig. 5c). The faster HSN setup seems to yield slightly higher outcomes for both CQAs compared to the default HRN setup, but overall comparable values were obtained for the degree of galactosylation and afucosylation.

**Fig. 6:**
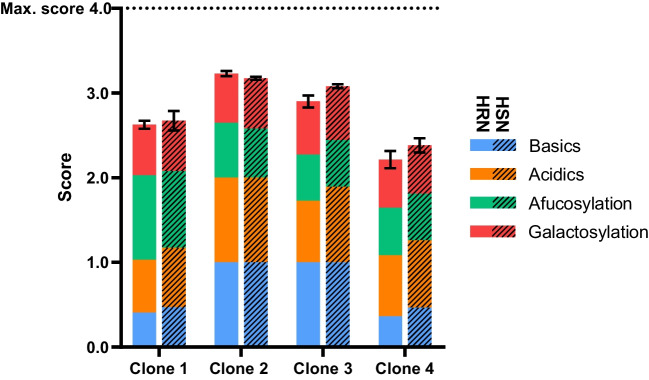
Bar chart depicting the cell line selection scores for the clone mAbs based on originator similarity in terms of afucosylation, galactosylation, basic variants, and acidic variants as measured using MCE-MS employing the HRN chip with an effective separation-channel gas pressure of 2.0 psi (solid bars) or the HSN chip employing an effective separation-channel gas pressure of 0.75 psi (striped bars) during analysis. The error bars indicate ± 1 standard deviation (*n* = 3). Scoring procedure is described in ref. [8]

To determine whether the HSN setup would identify the same high-quality clones as the default HRN setup, a cell line selection was performed according to a scoring procedure described previously. Figure S4 shows the relative differences between the clone mAbs and the originator for each CQA and MCE-MS setup, which were used to score the clones. As can be seen in Fig. 6 and Table S3, both setups result in comparable scores and consequently in the same ranking of the clone mAbs. In the context of this small-scale clone screening, clone 2 would be selected as the most biosimilar clone for both the HRN and the HSN setup.

To summarize, based on the analysis of mAb samples relevant to biosimilar clone screening, we conclude that the HSN chip together with the modified instrument provides essentially the same performance as the default HRN method, albeit at 3.3-fold shorter total analysis time. The HSN method theoretically allows running 314 injections per day, as opposed to 96 injections per day with the HRN method. Shorter analysis time thus results in increased instrument availability, which means that more biosimilar programs can be run on the same instrument at the same time compared to longer methods, ultimately leading to faster decision making. This increase in throughput together with MS identification can be beneficial for many stages in biotherapeutic drug development, such as extensive forced degradation studies, clone screenings, or large Design of Experiment (DoE) process development screenings. The higher throughput of the HSN setup means that larger amounts of samples need to be prepared. A sample preparation procedure for a full 96-well plate with mAb samples (consisting of protein A purification or buffer exchange) typically takes less than 2 h to purify, which is already sufficient for this method. The first 96 samples can be run while the next plate is prepared in parallel. Ideally, liquid handling robots would be essential to prepare the samples. For example, Luan et al*.* show both pipette tip- and batch-based protein A purification using a liquid handler, allowing up to 2000 mAbs to be purified per day [24]. Such techniques would increase the throughput of the overall analysis and reduce analyst’s hands-on time.

## Conclusion

Charge-variant analysis of mAbs by MCE-MS was considerably accelerated by implementing a microfluidic chip with a short separation channel (10 cm). Extra band broadening was effectively mitigated by modification of the MCE-MS system, allowing independent adjustment of the inlet pressure of the separation channel. When applying a relatively low pressure of 0.75 psi, the attained resolutions for NISTmAb were greatly improved for the short separation channel chip, achieving baseline separation between almost all charge variants. The performance of the new MCE-MS method was demonstrated by the analysis of mAb samples used for biosimilar clone selection. Similar separation, relative peak areas, and biosimilar scores were obtained as compared with a previously reported MCE-MS method, but in a significantly shorter time. The new method exhibited a 3.3-fold shorter total analysis time (including conditioning steps), which means that up to 314 samples could potentially be run in 24 h. An additional benefit of the modified MCE-MS system is that it allows for greater control of the separation on the chip, by tuning the gas pressures to a desired value. The modified MCE-MS system in conjunction with the short-channel chips has the potential to be employed in many stages of biotherapeutic development, enabling both rapid and information-rich analysis of e.g. early-stage clone samples or later stage process development samples.

## Supplementary Information

Below is the link to the electronic supplementary material.

Supplementary file1 (DOCX 147 KB)

## Data Availability

The data supporting the findings of this study are available from the corresponding author upon reasonable request, excluding the data concerning the mAb biosimilar samples, which are confidential due to the nature of the project.
