# Supplementary material for: Accelerating microfluidic capillary electrophoresis-mass spectrometry for charge-variant and glycoform analysis of intact monoclonal antibodies
Source: Anal Bioanal Chem. 2026 Feb 5;418(15):4775–85. doi: 10.1007/s00216-026-06374-9 (PMC13388436; doi:10.1007/s00216-026-06374-9)
Supplement: Supplementary file 1 — Supplementary file1 (DOCX 147 KB) [file 216_2026_6374_MOESM1_ESM.docx]

**Supporting information**

Accelerating microfluidic capillary electrophoresis-mass spectrometry for charge-variant and glycoform analysis of intact monoclonal antibodies

Ruben Cageling^a,b,c^, Erin A. Redman^d^, J. Scott Mellors^e^, Karin Lubbers-Geuijen^a^, Govert W. Somsen^b,c^ and Kevin Jooß^b,c*^

*^a^ Protein Purification & Characterization department, Polpharma Biologics Utrecht, Yalelaan 46, 3584 CM, Utrecht, The Netherlands ^b^ Division of BioAnalytical Chemistry, Department of Chemistry and Pharmaceutical Sciences, Vrije Universiteit Amsterdam, de Boelelaan 1085, 1081 HV Amsterdam, The Netherlands ^c^ Centre for Analytical Sciences Amsterdam (CASA), Amsterdam, The Netherlands ^d^* *Repligen Corporation, Morrisville, NC, USA
^e^* *Move Analytical LLC, Carrboro, NC, USA*

*Corresponding author: Kevin Jooß, address: Division of BioAnalytical Chemistry, Department of Chemistry and Pharmaceutical Sciences, Vrije Universiteit Amsterdam, de Boelelaan 1085, 1081 HV Amsterdam, The Netherlands; email address: k.jooss@vu.nl

**Table S1.** Influence of the effective gas pressure applied to the separation channel on the average (± 1 standard deviation; n = 3) migration time (t_m_) and Full Width at Half Maximum (FWHM) of the main and variant peaks observed during MCE-MS of NISTmAb using the HSN chip. N.D., Not determined due to overlap with the main peak.

| **Peak** | **Basic 2** | | **Basic 1** | | **Main** | | **Acidic** | |
| --- | --- | --- | --- | --- | --- | --- | --- | --- |
| **Effective gas pressure (psi)** | **t_m_ (min)** | **FWHM (min)** | **t_m_ (min)** | **FWHM (min)** | **t_m_ (min)** | **FWHM (min)** | **t_m_ (min)** | **FWHM (min)** |
| **2.0** | 1.331  ± 0.043 | 0.021  ± 0.002 | 1.373 ± 0.041 | 0.021  ± 0.003 | 1.422  ± 0.045 | 0.022  ± 0.002 | 1.463  ± 0.046 | N.D. |
| **1.75** | 1.340  ± 0.027 | 0.020 ± 0.001 | 1.381  ± 0.027 | 0.020  ± 0.001 | 1.428  ± 0.032 | 0.022  ± 0.000 | 1.475  ± 0.039 | N.D. |
| **1.5** | 1.373  ± 0.036 | 0.017  ± 0.002 | 1.418  ± 0.041 | 0.016  ± 0.000 | 1.467  ± 0.043 | 0.018  ± 0.002 | 1.510  ± 0.053 | N.D. |
| **1.25** | 1.409  ± 0.022 | 0.016  ± 0.000 | 1.454  ± 0.024 | 0.016  ± 0.001 | 1.503  ± 0.031 | 0.017  ± 0.000 | 0.029  ± 0.005 | 0.029  ± 0.005 |
| **1.0** | 1.482  ± 0.032 | 0.016  ± 0.001 | 1.533  ± 0.032 | 0.014  ± 0.001 | 1.586  ± 0.036 | 0.015  ± 0.000 | 0.030  ± 0.001 | 0.030  ± 0.001 |
| **0.75** | 1.594  ± 0.023 | 0.016  ± 0.002 | 1.649  ± 0.021 | 0.015  ± 0.001 | 1.711  ± 0.026 | 0.016  ± 0.001 | 0.035  ± 0.001 | 0.035  ± 0.001 |
| **0.5** | 1.587  ± 0.016 | 0.019  ± 0.005 | 1.651  ± 0.020 | 0.017  ± 0.003 | 1.718  ± 0.019 | 0.019  ± 0.003 | 0.037  ± 0.005 | 0.037  ± 0.005 |

**Table S2.** Influence of the effective gas pressure applied to the separation channel on the average (± 1 standard deviation; n = 3) plate numbers of Basic variant 1 (*N_B1_*) and the Main mAb (*N_main_*); Resolutions of peak pairs Basic variant 2 – Basic variant 1 (*R_S,B2-B1_*), Basic variant 1 – Main mAb (*R_S,B1-M_*) and Main mAb – Acidic variant (*R_S,M-A_*); Peak-to-valley ratio for the Main mAb and Acidic variant peaks (*p/v_M-A_*). N.D., Not determined due to overlap with the main peak.

| **Effective gas pressure (psi)** | **Plate number** | | **Resolution** | | | **p/v ratio** |
| --- | --- | --- | --- | --- | --- | --- |
|  | ***N_B1_*** | ***N_main_*** | ***R_S,B2-B1_*** | ***R_S,B1-M_*** | ***R_S,M-A_*** | ***p/v_M-A_*** |
| **2.0** | 24,423 ± 7,391 | 23,742 ± 5,035 | 1.17 ± 0.10 | 1.34 ± 0.18 | N.D. | 1.1 ± 0.2 |
| **1.75** | 27,896 ± 3,509 | 23,983 ± 452 | 1.23 ± 0.03 | 1.35 ± 0.15 | N.D. | 1.0 ± 0.1 |
| **1.5** | 41,777 ± 1,090 | 35,375 ± 5,454 | 1.60 ± 0.09 | 1.63 ± 0.09 | N.D. | 1.5 ± 0.5 |
| **1.25** | 48,438 ± 5,632 | 45,436 ± 2,260 | 1.71 ± 0.10 | 1.78 ± 0.28 | 1.33 ± 0.17 | 2.0 ± 0.6 |
| **1.0** | 63,052 ± 5,923 | 60,677 ± 4,769 | 1.96 ± 0.02 | 2.12 ± 0.13 | 1.36 ± 0.13 | 2.5 ± 0.5 |
| **0.75** | 66,961 ± 5,510 | 66,863 ± 5,451 | 2.10 ± 0.24 | 2.39 ± 0.15 | 1.46 ± 0.02 | 2.7 ± 0.2 |
| **0.5** | 56,966 ± 17,980 | 48,066 ± 13,873 | 2.14 ± 0.31 | 2.29 ± 0.37 | 1.49 ± 0.14 | 3.1 ± 0.4 |

 **Fig. S1** Normalized base peak electropherograms (BPEs) obtained with MCE-MS of NISTmAb using the HSN chip employing effective separation-channel gas pressures of 0.5 to 2.0 psi.

**Fig. S2** Bar chart depicting the average resolution between the main mAb and the acidic variant peak obtained for the four clone mAbs and an originator mAb using MCE-MS employing the HRN chip with an effective separation-channel gas pressure of 2.0 psi (blue bars) or the HSN chip with a separation-channel gas pressure of 0.75 psi during (orange bars) during analysis. The error bars indicate ± 1 standard deviation (n = 3).

**Figure S3.** Bar charts depicting the average fractional abundance (%) of the glycoforms detected for the four clone mAbs and originator mAb measured using MCE-MS employing the HRN chip employing an effective separation-channel gas pressure of 2.0 psi (blue bars) and the HSN chip employing an effective separation-channel gas pressure of 0.75 psi (orange bars) during analysis. The error bars indicate ± 1 standard deviation (n = 3). The red asterisk indicates that a glycoform was determined manually using EIEs. Note that the glycoforms G0F + G0-GlcNAc (found in clones 1, 2 and 4) and G1F + G1F (found in all samples) have the same mass as G0 + G0F-GlcNAc and G0F + G2F, respectively, and therefore, cannot be distinguished.

**Figure S4.** Plots showing the difference in percentage points (%) between each of the four clone mAbs and the originator mAb for acidic and basic variants, and afucosylation and galactosylation glycoforms, extracted from results obtained with MCE-MS instrument using the HRN chip (left column) or the HSN chip (right column). The differences were calculated by subtracting the average relative outcome of the originator from the relative outcome measured for each clone mAb. Error bars indicate the standard deviation (n=3). The green area depicts the originator range (average of the originator mAb triplicates ± 10%), the yellow area represents difference values that are below the originator range but still have a desirable value, and the red area depicts difference values outside the originator range.

**Table S3.** Average ranking scores (n = 3) for each clone mAb with regards to similarity to the originator sample based on MCE-MS results obtained using the HRN and HSN chip. A higher total score means a higher degree of similarity to the originator mAb.

| **Chip** | **Type** | **Clone 1** | **Clone 2** | **Clone 3** | **Clone 4** | **Scoring range** |
| --- | --- | --- | --- | --- | --- | --- |
| **HRN** | **Basics** | 0.41 | 1.00 | 1.00 | 0.36 | *0 - 1* |
|  | **Acidics** | 0.62 | 1.00 | 0.73 | 0.72 | *0 - 1* |
|  | **Afucosylation** | 1.00 | 0.65 | 0.54 | 0.56 | *0 - 1* |
|  | **Galactosylation** | 0.60 | 0.58 | 0.63 | 0.57 | *0 - 1* |
|  | **Total** | 2.62 | 3.23 | 2.90 | 2.21 | *0 - 4* |
|  | **Stdev** | 0.05 | 0.03 | 0.07 | 0.10 |  |
| **HSN** | **Basics** | 0.47 | 1.00 | 1.00 | 0.47 | *0 - 1* |
|  | **Acidics** | 0.70 | 1.00 | 0.89 | 0.79 | *0 - 1* |
|  | **Afucosylation** | 0.91 | 0.58 | 0.55 | 0.55 | *0 – 1* |
|  | **Galactosylation** | 0.60 | 0.59 | 0.64 | 0.57 | *0 - 1* |
|  | **Total** | 2.67 | 3.17 | 3.08 | 2.38 | *0 - 4* |
|  | **Stdev** | 0.12 | 0.02 | 0.02 | 0.08 |  |
